# Early diagnosis of HIV/aids infection: concept analysis

**DOI:** 10.1590/0034-7167-2022-0565

**Published:** 2023-08-07

**Authors:** Fernando Hiago da Silva Duarte, Silmara de Oliveira Silva, Bertha Cruz Enders, Ana Luisa Brandão de Carvalho Lira, Rodrigo Assis Neves Dantas, Daniele Vieira Dantas

**Affiliations:** I Universidade Federal do Rio Grande do Norte. Natal, Rio Grande do Norte, Brazil

**Keywords:** Acquired Immunodeficiency Syndrome, HIV Infections, Early Diagnosis, AIDS Serodiagnosis, Hospital Units., Síndrome de Inmunodeficiencia Adquirida, Infecciones por VIH, Diagnóstico Precoz, Serodiagnóstico del SIDA, Unidades Hospitalarias., Síndrome da Imunodeficiência Adquirida, Infecções por HIV, Diagnóstico Precoce, Sorodiagnóstico da AIDS, Unidades Hospitalares.

## Abstract

**Objectives:**

to analyze the concept of “early diagnosis of HIV/Aids infection” in light of Walker and Avant’s conceptual analysis model.

**Methods:**

concept analysis study based on the framework proposed by Walker and Avant, instrumented by a scoping review conducted in April 2022, following the recommendations of the Joanna Briggs Institute and checklist Preferred Reporting Items for Systematic reviews and Meta-Analyses extension for Scoping Reviews. The search was made in eight data sources, obtaining sixteen articles.

**Results:**

the study found homosexual intercourses, early examination, anti-HIV antibodies, CD4 count, and sexually transmitted infection as the main attributes of the concept. As antecedents: information, risky behavior, unprotected sexual relations, prevention, and access to the service. As main consequences: antiretroviral treatment, seroconversion, transmission, and consultations.

**Final Considerations:**

the study approached the circumstantial situations of the theme, its attributes, antecedents, and consequences, qualifying the work process based on knowledge of nursing practice.

## RESUMO

**Objetivos:**

analisar o conceito “diagnóstico precoce da infecção por HIV/aids” à luz do modelo de análise conceitual de Walker e Avant.

**Métodos:**

estudo de análise de conceito baseado no referencial proposto por Walker e Avant, instrumentalizado por uma revisão de escopo realizada em abril de 2022, seguindo as recomendações do Joanna Briggs Institute e *checklist Preferred Reporting Items for Systematic reviews and Meta-Analyses extension for Scoping Reviews*. A busca foi feita em oito fontes de dados, obtendo-se 16 artigos.

**Resultados:**

verificou-se como principais atributos do conceito: Relações sexuais homossexuais, Exame precoce, Anticorpos anti-HIV, Contagem de CD4 e Infecção sexualmente transmissível. Como antecedentes: Informação, Comportamento de risco, Relações sexuais desprotegidas, Prevenção e Acesso ao serviço. Como principais consequentes: Tratamento antirretroviral, Soroconversão, Transmissão e Consultas.

**Considerações Finais:**

o estudo aproximou as situações circunstanciais da temática, seus atributos, antecedentes e consequentes, qualificando o processo de trabalho fundamentado no conhecimento da prática de enfermagem.

## RESUMEN

**Objetivos:**

analizar concepto “diagnóstico precoz de la infección por VIH/SIDA” fundado en modelo de análisis conceptual de Walker y Avant.

**Métodos:**

estudio de análisis de concepto basado en referencial propuesto por Walker y Avant, instrumentalizado por revisión de ámbito realizada en abril de 2022, siguiendo las recomendaciones de Joanna Briggs Institute y *checklist Preferred Reporting Items for Systematic reviews and Meta-Analyses extension for Scoping Reviews*. Búsqueda hecha en ocho fuentes de datos, obteniendo 16 artículos.

**Resultados:**

verificado como principales atributos del concepto: Relaciones sexuales homosexuales, Examen precoz, Anticuerpos Anti-VIH, Recuento de CD4 e Infección sexualmente transmisible. Como antecedentes: Información, Conducta de riesgo, Relaciones sexuales sin protección, Prevención y Acceso al servicio. Como principales consecuentes: Tratamiento antirretroviral, Seroconversión, Transmisión y Consultas.

**Consideraciones Finales:**

el estudio acercó las situaciones circunstanciales de la temática, sus atributos, antecedentes y consecuentes, calificando el proceso de trabajo fundamentado en el conocimiento de la práctica de enfermería.

## INTRODUCTION

In the 1980s, acquired human immunodeficiency syndrome (Aids) was seen as a lethal disease, surrounded by social and moral pressures, contributing to the stigmatization of the condition and consequent discrimination. However, there is a change in society’s perception of human immunodeficiency virus (HIV) infection, which is linked to increased knowledge about the ways of transmission and treatment^(1-2)^.

Current estimates from the Pan American Health Organization (PAHO) point to HIV as a global public health problem, with more than 33 million deaths. In Latin America, new cases have increased by 21% since 2010, with about 120 thousand new people infected in 2019, the year that ended with about 38 million people living with HIV^(3)^.

In the general context, the advancement of therapeutic resources has provided patients with a better perspective and quality of life since care currently promotes the control of the disease and the change from the condition of a “terminal state” (as it was known a few decades ago) to the status of chronic disease, in which the individual manages to have balance in all its social aspects, health, and quality^(4)^.

On the other hand, the acute phase of HIV infection occurs in the first weeks, and the individual, during this period, becomes intensely infectious due to the high viral load in the body. Consequently, the clinical changes that arise during this process can trigger hospital admissions, and newly diagnosed patients need cautious assistance from a multidisciplinary team^(3-4)^.

Also, after the acute phase (at about nine weeks) the latency phase is established. The latter, in turn, is related to a transmission capacity dependent on viral load and is variable according to each organism. From there, over the years and according to the lifestyle of the infected, there is a direct relationship between the increase in viral load and the decrease in CD4+ cell levels, providing the emergence of timely infections and bringing the need for hospitalization^(2)^.

The clinical changes and symptoms of this process can trigger hospital admissions to specialized services in infectious diseases in newly diagnosed patients. Often, the diagnosis of HIV/Aids is revealed during an inpatient process, which triggers individual and collective impacts, such as feelings of denial, guilt, and anger. As a result, the period of hospitalization causes patients high levels of anxiety, along with the fear of death^(5)^.

In view of the above, it is necessary to analyze the concept of “early diagnosis of HIV/Aids infection” in hospitalized individuals. It favors the clarification of useful concepts for the practice of health care for this population; in addition, the conceptual analysis is directly interconnected with the evolution of nursing knowledge.

## OBJECTIVES

To analyze the concept of “early diagnosis of HIV/Aids infection” in light of Walker and Avant’s conceptual analysis model.

## METHODS

### Ethical aspects

The research was not submitted to the Research Ethics Committee because it was a concept analysis with literature search only.

### Study design

This study is based on the conceptual analysis model proposed by Walker and Avant^(6)^. The concept analysis aims to examine its structures and function, assisting in understanding the phenomena of a specific area of knowledge. This model comprises eight stages: selection of the concept; determination of the objectives of the conceptual analysis; identification of the possible uses of the concept; determination of the defining attributes; identification of the model case; identification of additional cases; identification of the antecedents and consequences of the concept; and definition of empirical references^(6)^.

As for the first stage, the concept selected was “early diagnosis of HIV/Aids infection” in hospitalized patients. For the second stage, as the objective of the conceptual analysis, it is necessary to contribute to the qualification of care in health units with general care and also in units specialized in the care of patients with infectious diseases.

Sequentially, the study investigated the possible identifications of the use of this concept. For this, it searched the scientific databases through a scoping review developed following the recommendations of the International Guide Preferred Reporting Items for Systematic reviews and Meta-Analyses extension for Scoping Reviews (PRISMA - ScR) and the Reviewers Manual^(7-8)^ from Joanna Briggs Institute (JBI), with research protocol registered in the Open Science Framework platform (https://osf.io/jnfz9/).

The study followed the steps proposed by the JBI for the elaboration of a scoping review: 1) development of the research question; 2) describing the inclusion and exclusion criteria and aligning them to the research question; 3) search planning, selection, extraction, and presentation of evidence; 4) search for evidence; 5) selection of evidence; 6) extraction of evidence; 7) analysis, presentation, and synthesis of the results^(8)^.

There were no publications with a similar objective to the review when consulting the platforms: International Prospective Register of Systematic Reviews (PROSPERO), Open Science Framework (OSF), the Cochrane Library, JBI Clinical Online Network of Evidence for Care and Therapeutics (COnNECT+) and Database of Abstracts of Reviews of Effects (DARE).

For the formulation of the research question, the study used the mnemonic PCC (population, concept, and context), according to the JBI. Thus, we defined: P-seropositive individuals with HIV/Aids; C - early diagnosis; C - hospital units. Based on this, the following question was constructed: what attributes, antecedents, and consequences of early diagnosis events in patients with HIV/Aids?

### Period and place of study

The literature searches were conducted in April 2022 by searching the collection in eight data sources (Chart 1). For the search and identification of the studies, the descriptors indexed in the Health Science descriptors (DeCS) and Medical Subject Headings (MeSH) to adapt the searches to Portuguese and English, of which were used, respectively: “*HIV*/HIV,” “*Síndrome da Imunodeficiência Adquirida*/Acquired Immunodeficiency Syndrome,” “*Vírus da Imunodeficiência Humana*/Human Immunodeficiency Virus,” “*Diagnóstico Precoce*/Early Diagnostic,” and “*Unidades Hospitalares*/Hospital Units.”

**Chart 1:** Search syntaxes used in data sources, Natal, Rio Grande do Norte, Brazil, 2022

Data sources	Search syntaxes
BDENF	(HIV OR “*Síndrome da imunodeficiência adquirida*” OR “*Vírus da Imunodeficiência Humana*”) AND (“*Diagnóstico precoce*”) AND (“*Unidades hospitalares*” OR “*serviços de saúde*”)
Gale Academic Onefile	*HIV OR Acquired Immunodeficiency Syndrome OR Human immunodeficiency virus AND* Keyword: *Early DiagnosisAND*Keyword: *Hospital Units OR Health Services*
Google Scholar	*(HIV OR “Acquired Immunodeficiency Syndrom” OR “Human immunodeficiency virus”) AND (“Early Diagnosis”) AND (“Hospital Units” OR “Health Services”)*
LILACS	HIV OR “*Síndrome da imunodeficiência adquirida*” OR “*Vírus da Imunodeficiência Humana*” [words] and “*Diagnóstico precoce*” [words] and “*Unidades hospitalares*” OR “*serviços de saúde*” [words]
MEDLINE / PubMed	*((HIV OR “Acquired Immunodeficiency Syndrome” OR “Human immunodeficiency virus”) AND (”Early Diagnosis“)) AND (“Hospital Units” OR “Health Services”)*
SciELO	(^*^(HIV OR “Acquired Immunodeficiency Syndrome” OR “Human immunodeficiency vírus”)) AND ((“Early Diagnosis”)) AND ((“Hospital Units” OR “Health Services”))
Scopus	*TITLE-ABS-KEY (hiv OR “acquired immunodeficiency syndrome” OR “human immunodeficiency vírus”) AND TITLE-ABS-KEY (“early diagnosis”) AND TITLE-ABS-KEY (“hospital units” OR “health services”)*
Web of Science	*TS=(HIV OR Acquired Immunodeficiency Syndrome OR Human immunodeficiency vírus) AND TS=(Early Diagnosis) AND TS=(Hospital Units OR Health Services)*

The search strategy was adapted according to the specificities of each source used. However, the research preserved the combinations between the descriptors, and did not add time and language restriction filters. The syntaxes discussed are in Chart 1.

### Population and sample

The research identified a total of 3,134 scientific articles among the data sources. After applying the inclusion and exclusion criteria, it selected 16 studies to compose the final sample of this study.

### Criteria of inclusion and exclusion

The research included publications available in full and free of charge in electronic media, scientific articles without the restriction of languages and time frame, and those that responded to the study objective. It excluded abstracts, letters to the editor, opinion articles, studies that escape the proposed theme, and duplicate records.

Access to data sources was through the Journal Portal of the Coordination for the improvement of Higher Education Personnel Foundation (CAPES), through remote access to the content of the Federated Academic Community (CAFe), a tool paid for by the Federal University of Rio Grande do Norte (UFRN).

The reverse search was conducted in the references of the selected studies to avoid exclusion of articles relevant to the theme that were not contemplated with the proposed crosses.

### Study protocol

Two researchers looked for the articles independently and simultaneously to perform an initial screening based on the reading of the titles, abstracts and subsequent evaluation of the inclusion criteria. Possible disagreements between the reviewers regarding inclusion in the study at any stage of development was resolved by discussion between the authors or through a third researcher consulted to read the material in full and proceed to the tiebreaker for the composition of the final sample. The research did not use any software to manage references or remove duplicates.

### Analysis of results

The selected studies were synthesized using two charts. In the first, according to the variables: identification (ID), country/year, the title of the article, type of study, and journal. In the second, depending on the essential elements, attributes, antecedents, and consequences.

Later, following the steps of the model proposed by Walker and Avant^(6)^, a model case and the reverse have been described. The presentation of the results concerning the objective of this work is descriptive through the discussion of the steps selected for the analysis of the concept of “early diagnosis of HIV/Aids infection” in hospitalized patients, fulfilling the steps of the framework used.

## RESULTS

**Figure 1:**
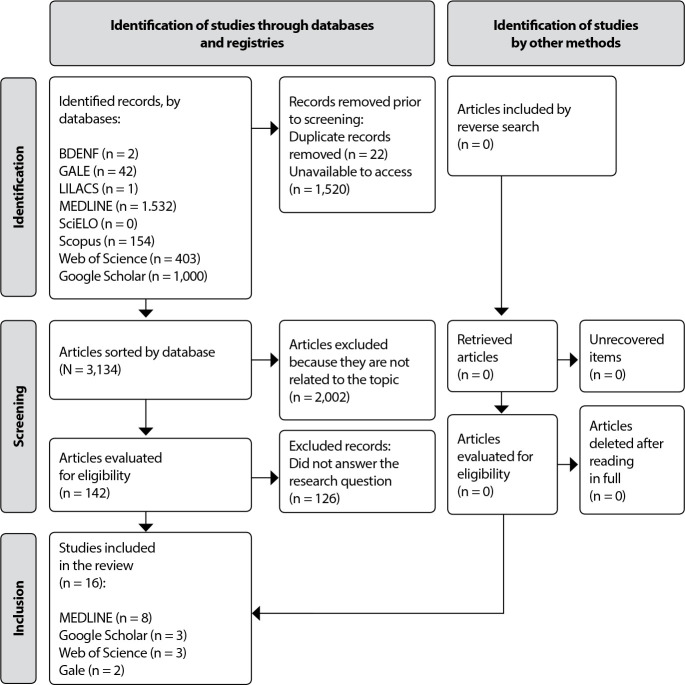
Prisma 2020 flowchart, adapted for scope review, Natal, Rio Grande do Norte, Brazil, 2022

The flowchart according to the phases of eligibility for selection and exclusion of findings is present in Figure 1.

After a survey of the data sources, the selected studies were identified in MEDLINE/PubMed (50%); Google Scholar and WOS (18.75% each); and Gale Academic Onefile (12.5%). The categorization of scientific articles is shown in Chart 2.

**Chart 2:** Categorization of the studies selected for the final sample (n = 16), Natal, Rio Grande do Norte, Brazil, 2022

^*^ID	Country / Year	Article title	Type of study
E1^(9)^	Sweden/2000	Diagnosis of primary HIV-1 infection and duration of follow-up after HIV exposure	Prospective study
E2^(10)^	Spain/2009	Increasing Early Diagnosis of HIV through Rapid Testing in a Street Outreach Program in Spain	Analytical study
E3^(11)^	United States of America (USA)/2010	Routine Opt-Out Rapid HIV Screening and Detection of HIV Infection in Emergency Department Patients	Prospective study
E4^(12)^	Spain/2011	Assessment of an outreach street-based HIV rapid testing programme as a strategy to promote early diagnosis: a comparison with two surveillance systems in Spain, 2008-2011	Comparative study
E5^(13)^	India/2012	Feasibility and Effectiveness of Provider Initiated HIV Testing and Counseling of TB Suspects in Vizianagaram District, South India	Cross-sectional study
E6^(14)^	USA/2014	Social Support as a Predictor of Early Diagnosis, Linkage, Retention, and Adherence to HIV Care: Results From The Steps Study	Prospective observational study
E7^(15)^	Kenya/2015	Stage of HIV presentation at initial clinic visit following a community-based HIV testing campaign in rural Kenya	Comparative study
E8^(16)^	USA/2017	Clinical and public health implications of acute and early HIV detection and treatment: a scoping review	Scope review
E9^(17)^	Australia/2018	Identifying missed clinical opportunities for the earlier diagnosis of HIV in Australia, a retrospective cohort data linkage study	Retrospective study
E10^(18)^	Spain/2018	The contribution of HIV point-of-care tests in early HIV diagnosis: community-based HIV testing monitoring in Catalonia, 1995 to 2018	Descriptive study
E11^(19)^	United States/2018	Antibody detection by agglutination-PCR (ADAP) enables early diagnosis of HIV infection by oral fluid analysis	Clinical trial
E12^(20)^	Spain/2019	*Evaluación de un programa de salud pública sobre diagnóstico de VIH con prueba rápida*	Descriptive study
E13^(21)^	USA/2020	Identifying and predicting longitudinal trajectories of care for people newly diagnosed with HIV in South Africa	Randomized controlled trial
E14^(22)^	Spain/2020	*Recomendaciones dirigidas a los servicios de urgências para el diagnóstico precoz de pacientes con sospecha de infección por VIH y su derivación para estúdio y seguimento*	Literature review
E15^(23)^	United Kingdom/ 2020	Evaluating the impact of post-trial implementation of RHIVA nurse-led HIV screening on HIV testing, diagnosis and earlier diagnosis in general practice in London, UK	Pragmatic study
E16^(24)^	Netherlands/ 2022	Testing and healthcare seeking behavior preceding HIV diagnosis among migrant and non-migrant individuals living in the Netherlands: Directions for early-case finding	Cross section

* *ID - Article identification.*

After analyzing the table, the research found that the years with the highest number of publications were 2018 and 2020, with three articles (18.75%) published each year. As for the country of publication, the United States and Spain had five (31.25%) each, followed by the United Kingdom with two (12.5%) publications, and the others, Sweden, the Netherlands, India, Kenya, and Australia, with one (6.25%) publication in each country.

After reading the selected articles in full, the researchers identified twelve attributes of the concept. They verified the frequency of attributes, which are words or expressions repeatedly present in the text, corresponding to the characteristics that express the concept^(6)^.

Some attributes and frequency in the articles stand out: homosexual intercourses (14%), early examination (12%), anti-HIV antibodies (9%), CD4 count and sexually transmitted infection (9 %), knowledge and heterosexual sexual relations (7%), lack of information (6%), early infection identification, self-care, and stigma (5%), the window period (4%).

In line with the steps of the model proposed by Walker and Avant^6^, the fifth and sixth stages of this study deal with the description of the model case and the opposite case.

### Early diagnosis model case

The model case makes a paradigmatic illustration of the concept exemplifying a case correlating the attributes. Below is a model case of the concept of early diagnosis in patients with HIV/Aids^(6)^.

“PJM, 28 years old, a male, completed high school, works as an administrative assistant in a private company, with admission to the emergency room presenting headache, fever, sore throat, and diarrhea for a few weeks. He reports having a homoaffective relationship, a steady partner, but with mutual agreement of an open relationship, in which they can relate sexually with other people. With your steady partner, oral and anal sex happens without the use of a condom; and, in casual sexual relations outside your relationship, oral and anal sex occurs with the use of a condom, most of the time. A rapid test was performed to detect HIV, with a positive result. PJM was referred to a reference unit, underwent complementary exams, and began his treatment with antiretroviral medications”.

### Opposite case of an early diagnosis

The opposite case illustrates the contrary of what the concept does not represent. Below is the opposite case of the concept of early diagnosis in patients with HIV/Aids^(6)^.

“PJM, 28 years old, a male, completed high school, works as an administrative assistant in a private company, with admission to the emergency room presenting headache, fever, sore throat, and diarrhea for a few weeks. Reports having a homoaffective relationship, a steady partner, but with mutual agreement of open relationship. Oral and anal sex happens without the use of a condom with his steady partner and also in casual sexual relations. The physician indicated a rapid test to detect HIV, but it was lacking in the unit. So, the patient was referred to a reference unit, but the schedules were suspended due to high demand. PJM improved his symptoms because he used the medications prescribed by the doctor who treated him; he continued to have unprotected sex inside and outside his relationship. After a few months, PJM was admitted to the emergency room with the same clinical symptoms, but with a worsening of the general condition and laboratory tests. There was a need for hospitalization, as he needed to treat acquired immunodeficiency syndrome (AIDS)”.

This fictitious opposite case presents disagreement with the essential attributes for identifying the concept of “early diagnosis” since the lack of information favors risk behaviors. In addition, Emergency Care Units (UPA) need to direct their efforts to keep rapid tests stocked to detect anti-HIV antibodies and effectively refer the patient with signs and symptoms of the infection to the nearest referral unit.

In the seventh stage of this study, researchers verified the antecedent and consequent elements of the concept of “early diagnosis”, being six antecedents and fifteen elements associated with the consequents, as described in Chart 3.

**Chart 3:** Frequency of antecedents and consequences of the concept “early diagnosis” in patients with HIV/Aids (n = 16), according to absolute and relative frequency, Natal, Rio Grande do Norte, Brazil, 2022

Essential elements	^*^ID	†F	‡%
**Antecedents**			
Risky behavior	E1, E2, E3, E4, E8, E9, E11, E12, E14, E15 e E16	19	12%
Information	E1, E2, E4, E13, E15 e E16	18	11%
Injectable drugs	E2, E3, E4, E10, E12, E14, E15 e E16	10	6%
Unprotected sexual relations	E1, E4, E8, E12, E14, E15 e E16	9	5,5%
Access to the service	E4, E5 e E9	4	2,5%
Prevention	E3 e E8	3	2%
**Consequential**			
Antiretroviral treatment	E1, E6, E7 E8, E9, E10 e E13	15	9,5%
Seroconversion	E1, E2, E8, E11 E12, E14 e E15	13	8%
Transmission	E2, E4, E7, E8, E9, E12, E14, E15 e E16	11	6,8%
Appointments	E6, E7, E8, E10 e E15	10	6%
Advice	E5, E7, E8, E15 e E16	10	6%
Promotion of quality of life	E1, E4, E5, E7 e E9	9	5,5%
Treatment	E1, E4, E5 e E9	8	5%
Antiretroviral drug	E1, E2, E4, E6 e E7	7	4,3%
Patient	E1, E2, E3, E4 e E8	6	3,8%
Social inclusion	E1, E2, E4, E6 e E7 e E10	6	3,8%
Chronic disease	E2, E4, E6 e E7	5	3,2%
Sexual conduct	E1, E2, E4, E6 e E7	5	3,2%
Prevention of opportunistic diseases	E1, E4, E5 e E9	4	2,5%
Examination	E2, E4 e E6	3	2%
Economic implications	E2, E4 e E9	3	2%

* *ID - article identification; †F - absolute frequency; ‡% - Relative frequency*

## DISCUSSION

The discussion is divided into two sessions by analyzing the attributes, antecedents and consequences of early diagnosis of HIV/Aids infection.

### Attributes of early diagnosis of HIV/Aids infection

Among the most evident attributes in this study are homosexual intercourses (14%), early examination (12%), anti-HIV antibodies, CD4 count, and sexually transmitted infections (9%). These identified elements demonstrate the characteristics of the studied phenomenon and the circumstances associated with the behavior that led the individual to a situation that could cause HIV infection.

Of the sixteen studies selected, eleven refer to homosexual intercourse as one of the factors associated with the risk of infection^(11-20,22,25)^.

At the beginning of the epidemic, Aids was characterized as a disease of gays, hemophiliacs, sex workers and intellectual people living in the capital. However, there are epidemiological changes evidenced and demonstrated through the Department of Informatics of the Unified Health System (DATASUS) and Information System on Notifiable Diseases (SINAN) database, where it shows a higher number of Aids cases in women, pauperization, internalization of the disease in the country^(26)^.

It is necessary to carry out actions that address the theme, especially in public places and on social media, because one of the biggest challenges to eradicate this endemic is associated with a lack of information; therefore, the more society is aware of the disease, ways of contagion and symptoms, the greater the chance that the infected individual will seek a health service^(12,18,20)^.

HIV infection can cause changes in the individual’s body and generate the possibility of developing specific symptoms from the acute to the advanced stage of Aids. Thus, the promotion of knowledge on the subject is essential since this public presents symptoms of the disease, has risk behavior, and does not perform post-exposure behaviors; and it is estimated that the average time between infection and the appearance of the first symptoms of the disease is around ten years^(8)^_._

Changes in the epidemiological profile of HIV and technological advances also require health professionals and responsible agencies to disseminate educational information and updates on strategies capable of promoting the teaching-learning process^(27)^.

### Antecedents and consequences of early diagnosis of HIV/Aids infection

The antecedents found in the studies demonstrate the events that precede the concept of early diagnosis and help to understand the context of patients newly diagnosed with HIV/Aids.

Among the antecedents, the study identified six elements with significant frequency: risk behavior (12%), information (11%), injecting drugs (6%), unprotected sexual relations (5%), access to the service (2.5%) and prevention (2%).

Regarding risk behavior, information, unprotected sexual relation, and injectable drugs, HIV - the cause of Aids, currently classified as a chronic communicable disease - can be found in bodily fluids or secretions such as blood, semen, vaginal secretions, and breast milk^(3)^.

It can be transmitted through unprotected sexual relation, biological accidents with sharps contaminated by the virus, especially in health institutions (hospitals, clinics, or laboratories), sharing syringes and needles, by blood transfusion; it can also happen vertically between the infected mother and the child during pregnancy, childbirth or breastfeeding^(2-3)^.

Aids was an acute condition, as it manifested itself quickly after diagnosis and, as there were no treatments available, it had high mortality rates. After a few decades, it is considered a chronic communicable disease due, above all, to three factors: advances in knowledge about the natural history of HIV infection; the possibility of monitoring the progression of the disease with the appearance of laboratory markers such as CD4 T and viral load tests; and advances in treatment with antiretrovirals^(28)^.

Nowadays, there is an increase in the number of individuals infected and living with HIV and able to have a good quality of life because, with the technological advancement and growth of the pharmaceutical industry, specific drugs have been developed to contain viral replication, with the use of antiretroviral therapy (ART)^(22)^. Early diagnosis is essential for the best prognosis for those who live virus^(29)^.

Regarding the consequential terms, the most prevalent were antiretroviral treatment, seroconversion, transmission, consultations, and counseling. Over time, there has been an evolution in both diagnosis and treatment for “people living with HIV” (PHIV), which contributes to the consequent terms evidenced in the studies as they follow the evolution of the phenomena involved with the theme.

Regarding the diagnosis, it can be done with complementary tests, such as rapid tests (RT); and also, through the screening immunoassay test - ELISA, with enzyme-linked immunosorbent assay, used in adolescents, adults, and children over 18 months of life. For confirmation of the diagnosis, Immunoblot, and Westerm Blot tests can be used or inline immunoassays, and molecular tests, the latter being able to identify the antigen, RNA, and pro-viral DNA of HIV. Such examinations are efficient and provide the diagnosis of acute and chronic infections, allowing treatment strategies to be accelerated^(25,27)^.

At the national level, mortality rates due to Aids show a significant decrease. PHIV, when effectively use medications, can establish strategies to improve and ensure their work activities, family life, and quality of life. In addition, the role of health professionals, especially nurses, in monitoring the patient about treatment failures is essential for successful patient adherence^(20)^.

In this expanded context of nursing practice, the care provided begins to focus not only on the individual but also on their family since professionals begin to understand that physical, emotional, and psychological changes can affect family members as everyone will have to organize and resize their lives and learn to live with the disease and the implications arising from it^(24)^.

In the case of attributes, antecedents, and consequences, it is relevant to emphasize that, as nursing care practice for people with HIV can be modified due to various contexts (hospital, home, specialized centers, as well as characteristics and needs of the patient), these conceptual elements may change. Therefore, it is imperative to develop other studies that will elucidate the concept of early diagnosis of HIV/Aids in the diversity of these contexts^(10,13,15)^.

The development of nursing knowledge represents new theoretical approaches and care strategies based on established theories, and theoretical frameworks strengthen practice by providing means to interpret nursing study objects, contributing to reflection and thus generating feedback between theory and practice^(16-17,19,22)^.

### Study limitations

One limitation was the use of only free-access articles, which may have reduced the sample size.

### Contributions to the fields of Nursing, Health, or Public Policy

The study promotes the understanding of the concept “early diagnosis of HIV/Aids infection,” contributing to professionals having a broad view of the factors associated with it. Thus, by understanding the phenomenon investigated, health professionals can develop welcoming strategies for patients who are diagnosed during the hospitalization process.

## FINAL CONSIDERATIONS

Concept analysis made it possible to clarify the concept of “early diagnosis of HIV/Aids infection,” demonstrating the phenomena involved with the theme throughout history. It may subsidize other studies since the concepts undergo modifications as health practices advance.

Thus, the study helps professionals understand the various difficulties involved in coping with HIV, social stigma, difficulties in coping with hospitalization processes, therapeutic failures and improving the quality of life of these patients.

Finally, this work represents an advance for the literature on the subject by synthesizing and analyzing the phenomenon of early diagnosis of HIV/Aids infection.
